# The Influence of Inflammatory and Nutritional Status on the Long-Term Outcomes in Advanced Stage Ovarian Cancer

**DOI:** 10.3390/cancers16142504

**Published:** 2024-07-10

**Authors:** Nicolae Bacalbasa, Sorin Petrea, Bogdan Gaspar, Lucian Pop, Valentin Varlas, Adrian Hasegan, Gabriel Gorecki, Cristina Martac, Marilena Stoian, Anca Zgura, Irina Balescu

**Affiliations:** 1 Department of Visceral Surgery, Center of Excellence in Translational Medicine “Fundeni” Clinical Institute, 022328 Bucharest, Romania; nicolae_bacalbasa@yahoo.ro; 2 Department of Surgery, “Carol Davila” University of Medicine and Pharmacy, 022328 Bucharest, Romania; bogdangaspar@yahoo.com; 3 Department of Surgery, “Ion Cantacuzino” Clinical Hospital, 022328 Bucharest, Romania; 4 Department of Visceral Surgery, “Floreasca” Clinical Emergency Hospital, 022328 Bucharest, Romania; 5 Department of Obstetrics and Gynecology, “Carol Davila” University of Medicine and Pharmacy, 022328 Bucharest, Romania; lucianpop@yahoo.com (L.P.); valentinvarlas@yahoo.com (V.V.); 6 Department of Obstetrics and Gynecology, National Institute of Mother and Child Care Alessandrescu-Rusescu, 022328 Bucharest, Romania; 7 Department of Obstetrics and Gynecology, “Filantropia” Clinical Hospital, 022328 Bucharest, Romania; 8 Department of Urology, Sibiu Emergency Hospital, Faculty of Medicine, University of Sibiu, 550169 Sibiu, Romania; adrianhasegan@yahoo.com; 9 Department of Anesthesia and Intensive Care, CF 2 Clinical Hospital, 022328 Bucharest, Romania; gabrielgorecki@yahoo.ro; 10 Faculty of Medicine, Titu Maiorescu University, 022328 Bucharest, Romania; 11 Department of Anesthesiology, Fundeni Clinical Hospital, 022328 Bucharest, Romania; cristinamartac@yahoo.com; 12 Department of Internal Medicine, “Carol Davila” University of Medicine and Pharmacy, 022328 Bucharest, Romania; marilenastoian@yahoo.com; 13 Department of Internal Medicine and Nephrology, Dr. Ion Cantacuzino Hospital, 022328 Bucharest, Romania; 14 Department of Medical Oncology, Oncological Institute Prof. Dr. Al. Trestioreanu, 022328 Bucharest, Romania; ancazgura@yahoo.com; 15 Department of Medical Oncology, “Carol Davila” University of Medicine and Pharmacy, 022328 Bucharest, Romania; 16 Department of Visceral Surgery, “Carol Davila” University of Medicine and Pharmacy, 022328 Bucharest, Romania; iri-na_balescu206@yahoo.com

**Keywords:** nutritional status, inflammatory status, ovarian cancer, long-term outcomes, debulking surgery

## Abstract

**Simple Summary:**

Achieving improved rates of overall survival remains a significant challenge in advanced-stage ovarian cancer; even if complete debulking is achieved, certain patients report poor results in terms of survival. Therefore, the aim of this paper is to investigate new prognostic markers that could point out which cases could benefit most from debulking surgery and which ones should be rather submitted to neoadjuvant therapies followed by debulking surgery.

**Abstract:**

Background: Despite improving surgical techniques and achieving more often complete debulking procedures, certain patients with advanced-stage ovarian cancer still have a very poor prognosis. The aim of the current paper is to investigate whether inflammatory and nutritional status can predict the long-term outcomes of ovarian cancer patients. Methods: A retrospective analysis of 57 cases diagnosed with advanced-stage ovarian cancer submitted to surgery as first intent therapy was carried out. In all cases, the preoperative status was determined by calculating the CRP/albumin ratio, as well as the Glasgow score, the modified Glasgow score and the prognostic nutritional index. Results: Patients presenting higher values of the CRP/albumin ratio, with a higher Glasgow score, modified Glasgow score and prognostic nutritional index (PNI), were more frequently associated with incomplete debulking surgery, a higher peritoneal carcinomatosis index and poorer overall survival (20 months versus 9 months for the CRP/albumin ratio *p* = 0.011, 42 versus 27 versus 12 months for the Glasgow score *p* = 0.042, 50 versus 19 versus 12 months for the modified Glasgow score, *p* = 0.001, and 54 months versus 21 months, *p* = 0.011 for the prognostic nutritional index). Conclusions: A strong relationship between the nutritional and inflammatory status in advanced-stage ovarian cancer seems to exist.

## 1. Introduction

Ovarian cancer ranks as the seventh most commonly diagnosed malignancy among women worldwide, with the highest incidence rates being reported in Eastern and Central Europe [1,2]. Therefore, it is estimated that a woman’s risk of developing ovarian cancer is 1 in 75, while the risk of dying due to this disease is 1 in 100 [3]. One of the most important problems regarding this pathology is the fact that almost 85% of cases are diagnosed in the advanced stages of the disease when the five-year survival rate decreases to 30% [4]. Despite the magnitude of the problem, the etiology of this malignancy as well as the prognostic factors, are not fully understood.

Although the golden standard in treating advanced-stage ovarian cancer remained debulking surgery to no residual disease, studies have shown that more than one-half of the patients will develop at least one recurrence within the next years, necessitating reoperation while others will die due to this disease in the next period [5,6,7]. Therefore, attention was focused on identifying the prognostic factors that might predict the risk of early recurrence and might provide a better identification of patients at risk to recur [8,9,10].

In this respect, particular interest was given to the impact of inflammatory status on the long-term outcomes of these patients, with a higher amount of cytokines, interleukins and tumor growth factors being frequently encountered in such cases. Therefore, it seems that the synthesis of inflammatory molecules generates a favorable microenvironment for the dissemination of tumoral cells, leading to the development of micrometastatic disease [11]. It has been widely demonstrated that cases presenting a more pronounced inflammatory condition are at risk of reporting poorer disease-free survival intervals and overall survival rates. Moreover, it seems that the administration of anti-inflammatory drugs might have a protective effect against carcinogenesis [12,13,14,15]. Meanwhile, it has been widely demonstrated that patients presenting an association between high inflammatory status and poor nutritional status have an even poorer prognosis in both benign and malignant conditions. For example, an elevated C-reactive protein/albumin (CRP/albumin) ratio has been traditionally associated with poor outcomes in septic patients, as well as in cases with hepatocellular, esophageal or gastric cancers [16,17]. Moreover, other scores taking into account both inflammatory and nutritional status, such as the Glasgow score, modified Glasgow score, or prognostic nutritional index (PNI), have been proposed as prognostic markers in different neoplastic processes, such as gastric, lung or breast cancer, and proved to have a prognostic value in such cases [18,19,20]. When it comes to the role of nutritional and inflammatory scores as predictive factors for ovarian cancer patients, conflicting results have been reported so far [21,22,23,24].

The aim of the current study was to analyze the outcomes of a group of patients submitted to primary cytoreductive surgery based on their preoperative nutrition and inflammation scores. Therefore the aim of the present paper was to investigate the prognostic value of different nutritional and inflammatory scores such as the CRP/albumin ratio, Glasgow score, modified Glasgow score, prognostic nutritional index (PNI) and systemic inflammatory index (SII) in order to identify those with the best predictive value for the long-term outcomes of ovarian cancer patients. Once such a prognostic tool is identified, it could be routinely used during the preoperative work up in order to identify cases who could benefit most from upfront debulking surgery. Once these protocols are implemented, an improvement in terms of survival is expected.

## 2. Materials and Methods

### 2.1. Study Population

Between 2014 and 2019, 73 patients with advanced-stage ovarian cancer were submitted to surgery at the “Ion Cantacuzino” Clinical Hospital in Bucharest, Romania. In all cases, debulking surgery to no residual disease was tempting and was the first therapeutic strategy. Patients with prior neoadjuvant chemotherapy were excluded from this study; therefore, we considered that administration of any neoadjuvant therapy might influence both the nutritional status as well as the inflammatory condition of the patient.

In all cases, clinical, laboratory and histopathological data were retrospectively reviewed, with preoperative blood tests being harvested one day before surgery. In order to increase the accuracy of the results, we excluded from the current study all patients with concurrent infections (13 patients—eight cases with a recent history of respiratory infections and five cases with urinary tract infections) as well as cases with a prior history of other neoplasia—one case with a previous history of colon cancer and two cases with prior history of hematological disorders. The final study group included 57 patients with a mean age of 56 years (range 25–83 years); in all cases, the preoperative complete blood count, CRP, albumin and protein levels were determined one day before surgery. In all cases, a classification according to the 2018 FIGO staging system was performed [25]. When it comes to the completeness of cytoreduction, complete debulking was considered if, at the end of the procedure, no visible residual disease was present, while incomplete cytoreduction was defined by the presence of macroscopic visible residual disease at the end of the procedure. The intraoperative extent of the lesions of peritoneal carcinomatosis was established by calculating the peritoneal carcinomatosis index based on Sugarbaker’s score. Therefore, the abdominal surface was divided into 12 regions, each region receiving a score between 0 and 3 according to the dimensions of the tumors as follows: 0 if no tumor was encountered; 1 if the largest tumor was up to 0.65 cm; 2 if the largest tumor was up to 5 cm; and 3 if the largest tumor measured more than 5 cm [26]. When it comes to the histopathological classification, the classification was conducted according to the WHO 2020 protocol; moreover, the degree of differentiation of each lesion was also determined during the histopathological studies [27]. The early postoperative morbidity rate was classified according to the Dindo–Clavien scale, while the long-term outcomes were evaluated based on the progression-free and survival rates. In all cases, the long-term follow-up was performed every three months within the first two years and every six months until the five-year mark and included laboratory tests—including the CA125 dosage—by using an electrochemiluminescence immunoassay analyzer with a cut-off value of 35 U/dL [28], thorax computed tomography and abdominal and pelvic magnetic resonance imaging. When it comes to adjuvant chemotherapy, in all cases, the standard therapeutic regimen consisting of taxanes and platinum-based salts was administrated

### 2.2. Study Design

**Figure 1:**
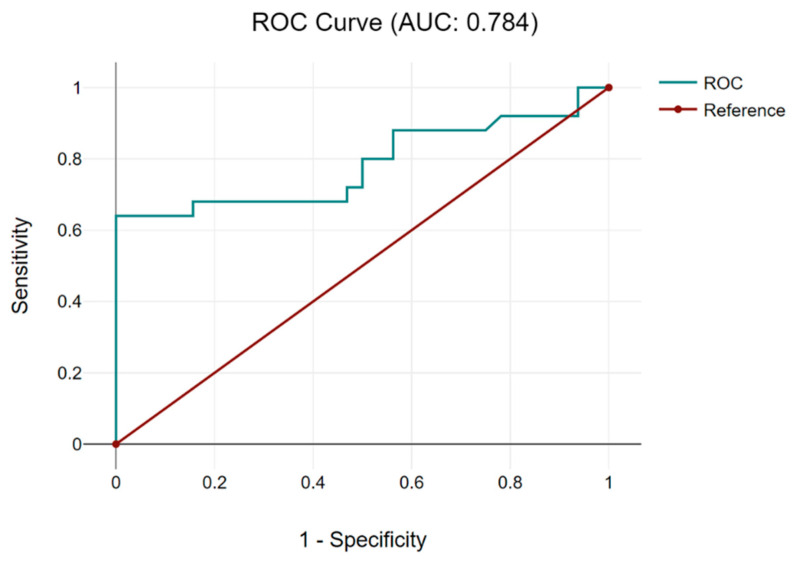
The ROC curve for the CRP/albumin ratio establishes a cut-off value of 0.05. Based on the cut-off value of 0.05 for the CRP/albumin ratio, patients were further classified into two categories: cases with a CRP/albumin ratio < 0.05—9 patients—and cases with a CRP/albumin ratio > 0.05—48 cases.

The receiver operating characteristic (ROC) curve was used with the Youden index [maximum (sensitivity + specificity-1)], a cut-off value of the CRP/albumin ratio in order to predict the achievement of complete debulking surgery, being determined at 0.05 (with a sensibility of 0.8, 1-specificity of 0.75 and, respectively, with an area under the curve of 0.784) (Figure 1).

The next investigated parameters were represented by the Glasgow score, the modified Glasgow score and the PNI. The Glasgow score and the modified Glasgow score were based on the preoperative values of the CRP and, respectively, albumin levels; in both cases, a cut-off value of 10 mg/L was established for the CRP and, respectively, 35 g/L for albumin. According to these values, the patients were scored with 0 points if the CRP < 10 mg/L and, respectively, albumin > 35 g/L in both scores; 1 point if the CRP < 10 mg/dL and, respectively, albuminPNI was defined accordingly to the preoperative values of the CRP, albumin and lymphocyte count [29,30,31,32]. The scoring system for the Glasgow score and the modified Glasgow score are presented in Table 1. Meanwhile, when it comes to the PNI, it was defined as albumin (g/L) + 5 × total lymphocyte count × 109/L; a value of 0 is assessed if the PNI > 48 while patients with a PNI < 48 were scored with 1 point. In order to assess the inflammation status, the systemic inflammatory index (defined as neutrophils × platelets/lymphocytes) was used.

### 2.3. Statistical Analysis

Statistical analyses consisted of a Chi-square test for the categorical variables, a Student’s *t*-test for continuous variables, a Cox proportional hazard model for univariate analysis and a multivariate Cox proportional hazard model for multivariate analysis.

A *p*-value lower than 0.05 was considered statistically significant. The long-term outcomes were investigated by analyzing the disease-free and overall survival rates. The disease-free survival interval was defined as the period between initial surgery and the date of relapse, while the overall survival period was defined as the interval between the date of initial surgery and the date of cancer-related death. The median disease-free and overall survivals were obtained by performing a Kaplan–Meier analysis and were compared by using the log-rank test. A multivariate linear logistic regression was performed to assess the relation between disease-free survival and, respectively, the overall survival rate and different nutritional scores.

All data were analyzed by using the SPSS statistical software version 18.0 (SPSS Inc. Chicago, IL, USA). When it comes to the normal distribution tests, the mean and standard deviation were used.

## 3. Results

The mean age at the time of entering the study among the entire group was 56.4 years (range 25–83 years), while the FIGO stage was IIIC in 51 cases and, respectively, IV in 6 cases. During the preoperative workup, the mean value of CA 125 was 2892 U/dL (range 78–14,535 U/dL, standard deviation = 3707, 95%, confidence interval = 1930.18; 3854.98). The mean values of CRP and albumin, respectively, were 11 g/dL (range 2–21 g/dL, standard deviation = 8.62, 95% confidence interval = 8.78; 13.30) and 3.2 g/dL, respectively (range 1.6–4.4 g/dL, standard deviation = 0.85, 95% confidence interval = 3.03; 3.47). The mean CRP/albumin ratio was 0.41 (range 0.02–1.56, standard deviation = 0.4, 95% confidence interval 0.32; 0.52). The relation between the CRP/albumin ratio values and clinicopathological and postoperative findings is presented in the table below.

As can be observed from Table 2, there was no statistically significant relation between the preoperative CRP/alb ratio and age or associated comorbidities; however, patients with higher values of CRP/alb ratio were usually older and associated with more frequently concurrent diseases when compared to cases with a CRP/alb ratio < 0.05. The most commonly associated comorbidities were represented by arterial hypertension in eight cases, followed by cardiac congestion in four cases and diabetes mellitus in three cases; other encountered comorbidities were represented by hypothyroidism, asthma, depression and scleroemphysema. As it can be observed, none of the cases presented inflammatory systemic diseases or metachronous neoplastic processes, which might otherwise influence the values of the inflammatory and nutritional scores. Interestingly, although there was no significant relation between the preoperative CRP/alb ratio and the initial FIGO stage, cases with higher values of this parameter presented a higher value in the peritoneal carcinomatosis index (PCI) (with a marginal statistic significance of *p* = 0.051). This finding enables us to consider that a higher value of the CRP/albumin ratio was associated with a higher tumoral burden, demonstrated by a larger extent of the peritoneal lesions. Meanwhile, higher values were also associated with non-serous histology, with a significantly higher preoperative level of CA 125 (2825 versus 1142 U/dL, *p* = 0.003) as well as with a higher amount of ascites (3561 mL versus 1292 mL, *p* ≤ 0.001) while the maximum diameter of the tumor and the lymph node status were not correlated in a statistically significant manner with the preoperative CRP/albumin ratio. Interestingly, the differentiation degree of the lesions (diagnosed during the histopathological studies) was not influenced in a statistically significant manner by the preoperative value of the CRP/albumin ratio. All these data suggest the fact that this parameter was rather correlated with the presence of massive peritoneal dissemination, which also explains the higher amount of ascites and the higher values of CA125 levels.

When it comes to the relation between preoperative CRP/alb ratio and other inflammatory indexes, other interesting correlations were observed. Therefore, according to the data presented in Table 2, cases with a higher CRP/alb ratio were associated with a significant proinflammatory status, which was demonstrated by significantly higher values of NLR, PLR, MLR or SII. Meanwhile, as expected, patients with a more important inflammatory status are also associated with a lower preoperative level of hemoglobin (this aspect is explained through the fact that neoplasia and proinflammatory status are usually associated with a higher rate of anemia) (13.1 g/dL versus 11.6 g/dL, *p* = 0.015).

As for the intraoperative details regarding the extent of the surgical procedure in the upper abdomen, there was no statistically significant difference between the need to extend the procedure in the upper abdomen among cases with higher and, respectively, lower CRP/alb levels. This fact underlines that the extent in the upper abdomen is rather a sign of a longer period of evolution of the disease than of a more aggressive biology of the tumor. This aspect can also be argued by the fact that there was no relation between the preoperative level of CRP/alb ratio and the degree of differentiation observed in the histopathological studies (*p* = 0.098).

**Figure 2:**
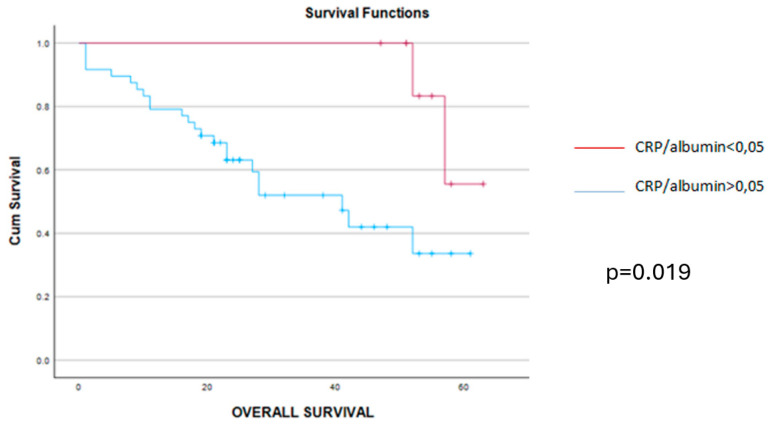
The mean overall survival was 9.36 months (confidence interval 4–11 months) for cases with CRP/alb > 0.05 (blue line) and 20.49 months (confidence interval 22–26 months) for cases with CRP/alb < 0.05 (red line) while the median overall survival was 8 months for cases with CRP/alb > 0.05 (blue line) and 26 months for those with CRP/alb < 0.05 (red line), *p* = 0.019.

**Figure 3:**
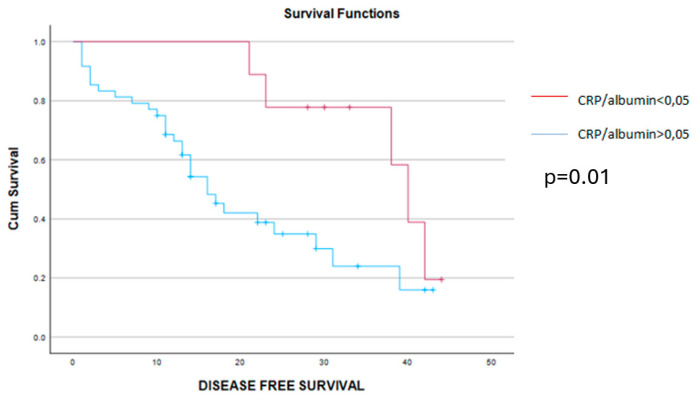
The mean disease-free survival interval was 6 months for cases with CRP/alb > 0.05 (blue line) and 14 months for cases with CRP/alb < 0.05 (red line), *p* = 0.01.

When analyzing the early postoperative complications, they were encountered in 16 cases, all of them being encountered among cases with higher values of the CRP/albumin ratio, *p* = 0.049. As for the long-term outcomes, cases with a lower preoperative value of the CRP/albumin ratio had a significantly better overall survival when compared to cases with higher values of this parameter (9 months versus 20.49 months, *p* = 0.019) (Figure 2). Meanwhile, when analyzing the impact of this parameter on the disease-free interval, the mean disease-free survival rate was 14 months among cases with lower values of the CRP/albumin ratio and 6 months for the others (*p* = 0.011) (Figure 3).

We went further with the analysis of the possible relation between nutritional and inflammatory status, and we investigated the distribution of the Glasgow score, modified Glasgow score and nutritional prognostic index. Therefore, according to the Glasgow scale, there were 19 patients who scored with 0 points, 21 patients who scored with 1 point and 17 cases who scored with 2 points. According to the modified Glasgow score, there were 32 patients who scored 0 points, 8 cases who scored 1 point, and 17 cases who scored 2 points, while according to the nutritional prognostic index (PNI), there were 39 cases with a PNI < 48 who scored with 1 point and 18 cases with a PNI > 48 who were scored with 0 points.

The analysis of different preoperative, intraoperative and postoperative parameters on different prognostic groups according to the three scores are presented in the tables below.

As can be observed from Table 3, there was a significant association between the Glasgow score and the other laboratory parameters, which provide information regarding the inflammatory and nutritional status of these patients, demonstrating, therefore, the strong relation between these two variables (defining the nutritional and inflammatory status). Meanwhile, patients with lower GS scores tended to be significantly younger, had a lower tumoral burden (demonstrated by significantly lower levels of CA125 and ascites) and were associated less frequently with lower levels of hemoglobin when compared to cases with higher GS values. As expected, complete cytoreduction was more frequently reported among cases with lower GS values—this fact resulted in significantly improved long-term outcomes. Therefore, both disease-free and overall survival were significantly higher among cases with GS = 0 when compared to cases with GS = 1 or 2.

**Figure 4:**
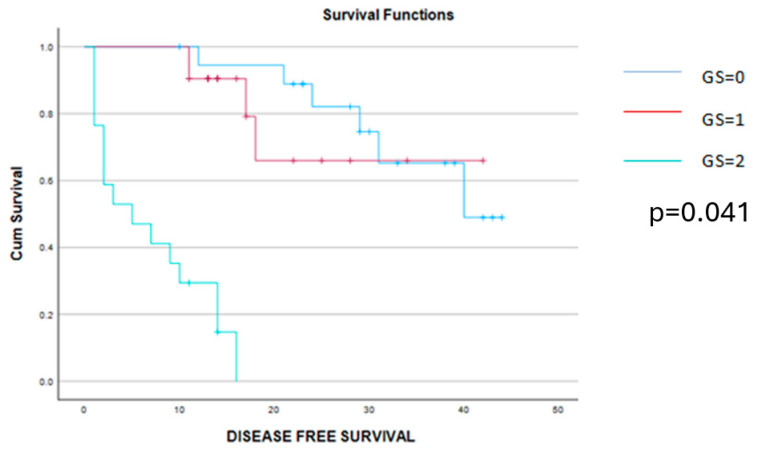
Kaplan–Meier survival curves demonstrating a significant benefit of disease-free survival among patients scored with 0 or 1 point on the Glasgow score when compared to those with a Glasgow score of 2 points.

**Figure 5:**
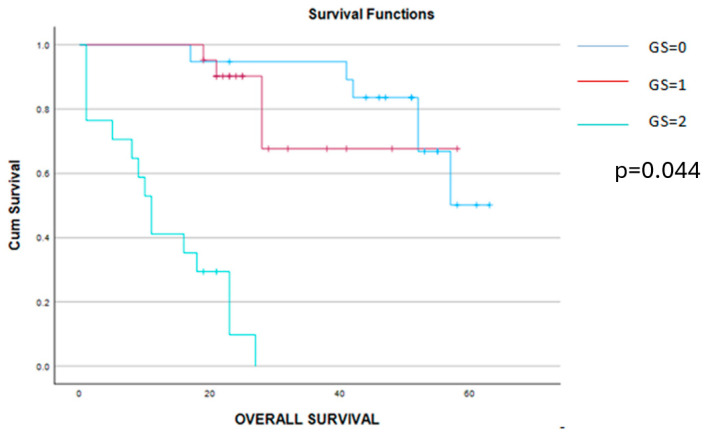
Kaplan–Meier survival curves demonstrating a significant benefit of the overall survival among patients scored 0 or 1 point when compared to those with a Glasgow score of 2 points.

The Kaplan–Meier disease-free survival and overall survival curves are presented in the Figure 4 and Figure 5.

The analysis of the impact of the modified Glasgow (MGS) score on the perioperative factors is shown in Table 4. Similarly to the results obtained when analyzing the GS values, patients presenting lower MGS values tended to be younger, to have a lower tumoral burden (expressed through a lower value of CA125 levels and of ascites volume), as well as lower inflammatory status and better nutritional status.

**Figure 6:**
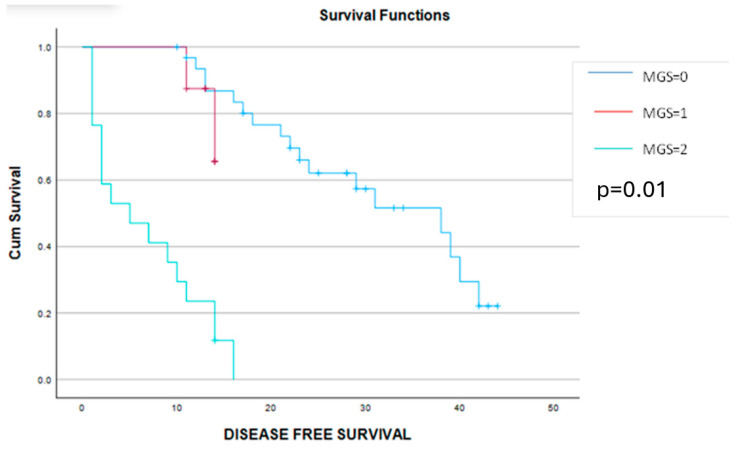
Kaplan–Meier graphic showing the disease-free survival curves for patients with MGS = 0 (blue line), MGS = 1 (red line) and MGS = 2 (green line) and demonstrating a significant benefit in terms of progression-free survival for cases with MGS 0 or 1 (30 months versus 13 months versus 6 months, *p* = 0.01).

**Figure 7:**
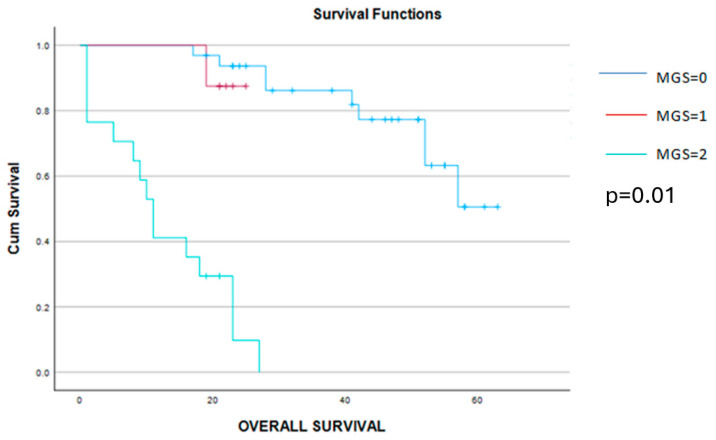
Kaplan–Meier graphic showing a significant benefit in terms of survival for patients with MGS 0 and 1 when compared to cases with MGS = 2 (50 months versus 19 months versus 12 months, *p* = 0.01).

This time, a significant correlation was found between the MGS, CA 125, NLT, MLR, SII and CRP/albumin ratio, while no statistical significance could be established between the MGS values and the value of preoperative hemoglobin. However, cases with 0 points, according to the MGS, had a slightly higher value of preoperative hemoglobin when compared to cases with 1 or 2 points with the MGS. When it comes to the long-term outcomes, cases with a lower MGS, patients had a significantly better outcome in terms of progression-free and overall survival when compared to cases with a higher MGS). Therefore, the mean disease-free survival interval was 31 months for cases with a MGS = 0, 13 months for a MGS = 1, and, respectively, only 6 months for cases with a MGS = 2. Meanwhile, when determining the mean overall survival rates for the three groups, this value decreased from 50 months (in cases with MGS = 0) to 19 months (in cases with a MGS = 1) and, respectively, 12 months (in cases with a MGS = 2) (Figure 6 and Figure 7).

The relation between different perioperative parameters and the PNI is presented in Table 5.

As can be observed from the abovementioned table, patients who scored 0 points with the PNI tended to be younger and had fewer associated comorbidities when compared to cases with a higher PNI. Although there was no statistical significance between the PNI and FIGO stage, patients with a PNI = 0 were usually diagnosed with less extended lesions, with lower CA125 levels, with lower volumes of ascites and with a lower value of the PCI. Although a positive relation failed to be demonstrated between the PNI and histopathological subtype, it seems that well-differentiated lesions, with no lymph node metastases, were usually associated with a higher PNI score. In our opinion, the relation between PNI values and lymph node metastases can be explained through the number of circulating lymphocytes. Therefore, cases with higher PNI values also had higher preoperative values of lymphocytes, and due to this reason, usually had less frequent lymphatic metastases.

Similarly to the other nutritional and inflammation scores, patients with a lower PNI were usually associated with higher NLR, PLR, MLR and SII values as well as with a poorer nutritional level (defined through a lower level of total serum proteins—7.19 g/dL versus 5.49 g/dL, *p* = 0.018). Surprisingly, a positive correlation between the PNI and preoperative levels of hemoglobin failed to be demonstrated this time; this aspect can be explained through the fact that in neoplastic diseases, the presence of anemia can be explained through multiple mechanisms. When it comes to the necessity of performing upper abdominal resections, it was similar between the two groups. This aspect came to underline, once again, that the upper abdominal involvement is probably the expression of a longer evolution of the disease and not of a poorer biology of the tumor, which might induce host immunosuppression and progression of the disease.

As expected, cases with a poorer nutritional status and with a poorer protective level (defined through a lower level of circulating lymphocytes) developed postoperative complications more frequently; moreover, we came to demonstrate that all severe complications, as well as all cases of early postoperative death (within the first month), were encountered among cases with lower PNI values.

**Figure 8:**
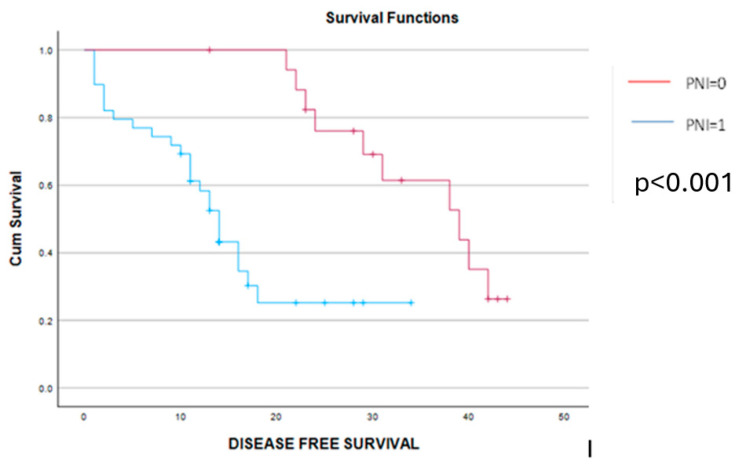
Kaplan–Meier disease-free survival curves demonstrate a significant benefit among cases with PNI = 0 (red line) versus cases with PNI = 1 (blue line). The mean disease-free survival interval was 35 months among cases with preoperative PNI = 0 and, respectively, 11 months for cases with preoperative PNI = 1 (*p* < 0.001).

**Figure 9:**
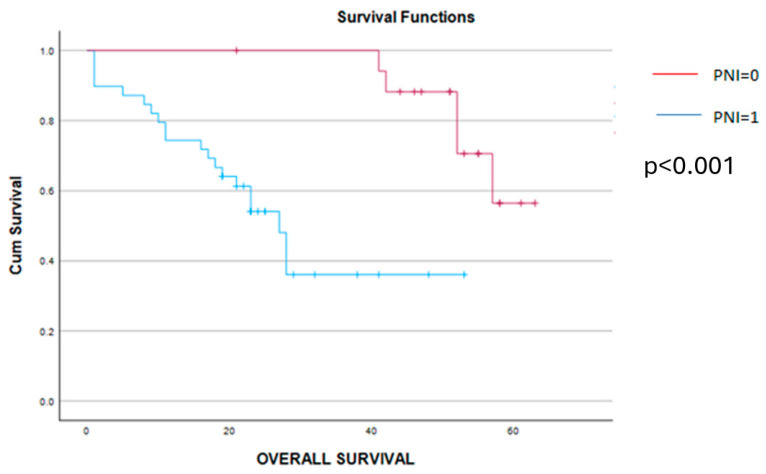
Kaplan–Meier overall survival curves demonstrate a significant benefit in terms of survival for cases with PNI = 0 (red line) versus cases with PNI = 1 (blue line). Therefore, the overall survival rate was 54 months for cases with preoperative PNI = 0 and, respectively, 21 months for cases with preoperative PNI = 1 (*p* < 0.001).

As for the long-term outcomes, a significant benefit in terms of disease-free and overall survival was obtained in cases with a PNI = 0. Therefore, patients with a PNI = 0 reported a mean disease-free survival interval of 35 months and, respectively, an overall survival rate of 54 months, while cases with a preoperative PNI = 1 reported a mean disease-free survival interval of 11 months and, respectively, an overall survival rate of 21 months (*p* < 0.001 in both cases) (Figure 8 and Figure 9). The log-rank Kaplan–Meier survival curves are presented in the figures below.

When analyzing the data obtained after stratifying ovarian cancer patients according to the four nutritional indexes, we observed that higher values of the CRP/albumin ratio, GS, MGS, and, respectively, higher PNI values, were also associated with the presence of a severe systemic inflammatory status (defined by a significantly higher level of NLR, PLR, MLR and, respectively, SII). Interestingly, when studying the possible correlation between the preoperative levels of hemoglobin and the different proposed scores, a positive relation could be established only between this parameter and the preoperative values of the CRP/albumin ratio and GS, respectively. This aspect might be explained through the fact that anemia can be explained through multiple mechanisms in advanced-stage ovarian cancer, not only by an exacerbated inflammatory status. Another interesting aspect that should be discussed is the correlation between the CRP/albumin ratio and, respectively, PNI and the rates of lymph node involvement. Although a higher level of CRP/albumin ratio was associated more frequently with the presence of positive lymph nodes, this fact did not reach statistical significance; however, the presence of lymph node metastases was positively related to the preoperative values of the PNI. In our opinion, this aspect can be explained through the fact that a lower number of circulating lymphocytes is expected to be associated with poorer host defense against neoplastic dissemination, poorer PNI values, and, respectively, higher rates of lymph node metastases. As for the peritoneal extent of the disease, in all cases, a poorer nutritional status and, respectively, a more pronounced inflammatory status were significantly associated with a higher tumoral burden (defined through a significantly higher amount of ascites); moreover, when analyzing the distribution of the PCI among the different groups, a higher value was statistically significant associated with a higher CRP/albumin ratio and, respectively, with a higher PNI value.

A multivariate logistic regression was performed to assess the relation between disease-free survival and, respectively, overall survival rate and SG and, respectively, the PNI. Data were checked for multicollinearity with the Belsley–Kuh–Welsch technique. Heteroskedasticity and normality of residuals were assessed, respectively, by the Breusch–Pagan test and the Shapiro–Wilk test. A *p*-value of <0.05 was considered statistically significant. In the multivariate analysis, SG = 0 (β = 8.82, [2.03; 15.61], *p* = 0.011) was associated with higher values of disease-free survival, while SG = 2 (β = −8.59, [−14.97; −2.21], *p* = 0.009) was associated with lower values of this parameter. The preoperative PNI values (β = 0.26, [−0.12; 0.64], *p* = 0.173) were not associated with disease-free survival. When it comes to the overall survival rates, SG = 0 (β = 16.9, [8.3; 25.5], *p* < 0.001) was associated with higher values of overall survival, while SG = 2 (β = −13.0, [−21.08; −4.91], *p* = 0.002) was associated with lower values of this parameter. Meanwhile, the PNI values (β = 0.31, [−0.17; 0.79], *p* = 0.199) were not associated with the overall survival rate.

## 4. Discussions

Data obtained in this paper demonstrate the fact that between the nutritional and inflammatory status, a strong relation can be established, which further seems to influence the long-term prognosis of ovarian cancer patients. In our opinion, the great advantage of these scores is represented by the fact that they can be easily determined during the preoperative period based on the routine blood tests that are harvested before any surgical procedure. Once these scores are determined, it seems that they can be used as predictive tools in order to identify cases who could benefit most from an aggressive surgical approach, as well as cases in which per primam surgery should be rather replaced by neoadjuvant chemotherapy [33,34,35]. As it can be observed from the data we showed in this paper, cases with poorer nutritional status in association with a higher inflammatory status seem to have a significantly higher risk of developing life-threatening complications or experiencing a poorer long-term outcome (translated through a poorer disease-free and, respectively, overall survival). In our study, the most relevant differences were observed when studying the impact of the CRP/albumin ratio on the postoperative outcomes; although the other investigated scores were also associated with the risk of developing postoperative complications as well as with a poorer long-term outcome, the statistical significance was lower.

Meanwhile, another aspect that should be pointed out is the one related to the correlation between nutritional scores, CA125 and other inflammatory scores. According to our results, a statistically significant difference was observed when analyzing the relationship between these parameters. Moreover, when investigating the possible association between these parameters and the upper abdominal extent, we could not find any positive correlation. These data demonstrate once again the fact that an upper abdominal extension should be considered as a sign of a longer evolution of the disease and not of a poorer biology of the tumor. However, in our opinion, particular interest should be given to the PNI—the only nutritional score that takes into account the host’s capacity to fight against cancer dissemination (through the number of circulating lymphocytes) as well as the nutritional status itself, defined through the systemic level of serum albumin [36,37,38,39,40].

Initially, the PNI was proposed by Onodera et al. in 1984 in order to better identify cases at risk of developing severe postoperative complications after gastrointestinal surgery [37]. More recently, this parameter was also included as a prognostic marker for lung cancer and gynecological-tract cancers, being able to predict both the risk of early postoperative complications as well as the long-term oncological evolution of such cases [41]. Interestingly, when it came to ovarian cancer, conflicting results have been reported so far [29,30,42]. Initially, the prognostic value of the PNI in ovarian cancer has been considered a controversial one, especially due to the low number of cases initially included in such analyses and the high diversity of the included cases. Therefore, one of the first studies that came to demonstrate the predictive value of the PNI, even when it comes to early-stage ovarian cancer, was published by Yoshikawa et al. in 2020 and included 82 patients with FIGO stage I–II ovarian cancer. The study demonstrated that a lower PNI value is associated with a better outcome in terms of overall survival in both univariate and multivariate analyses. Although the disease-free survival between the two groups was similar, the post-recurrence survival interval was significantly higher among cases with a lower PNI value [29]. Similarly to our results, Feng et al. demonstrated that the preoperative value of the PNI is strongly associated with the preoperative values of CA125, with the amount of ascites, with the risk of incomplete debulking and with the long-term outcomes. However, it seems that this parameter can also have a positive predictive value for identifying platinum-resistant cases [34]. One of the largest studies that came to demonstrate the positive predictive value of the PNI in ovarian cancer patients comes from Dai et al. and included 2050 patients from six studies. Interestingly, this meta-analysis included five studies conducted in China and one study conducted in Japan and demonstrated that the most important influence of the PNI on long-term outcomes was observed in the studies conducted in China. Overall, this meta-analysis underlined the fact that cases with a PNI = 1 were associated with shorter overall survival, worse clinicopathological features, a more advanced FIGO stage, higher amounts of ascites, larger residual tumors and a poorer response to chemotherapy. However, the authors underlined the fact that, although no restriction was posed in terms of race and country, all eligible studies originated from China and Japan and considered that this fact might reduce the accuracy of the results [31].

When it comes to the CRP/albumin ratio, we should not omit the fact that CRP represents an acute phase protein that is mainly synthesized by hepatocytes and which is strongly related to the level of systemic inflammation, the capacity of angiogenesis and the synthesis of interleukins and growth factors, which further promote tumoral proliferation and dissemination [32,43]. Meanwhile, albumin represents another product of the synthesis of hepatocytes, which usually reports lower levels in the presence of severe inflammatory or neoplastic processes.

In this respect, we should mention the fact that the studies published so far have demonstrated that the CRP/albumin ratio represents an important parameter in order to predict the long-term outcomes in other malignancies, such as hepatocellular carcinoma, esophageal or gastric carcinoma [29,44,45]. When talking about ovarian cancer, we should underline the fact that elevated serum levels of CRP are to be expected due to the presence of chronic inflammation, which is caused by the progression of the malignant disease. Meanwhile, malnutrition, which is constantly present in such cases, is translated through a low serum level of albumin [46].

Therefore, it is easy to understand the fact that the CRP/albumin ratio is considered a significant prognostic factor for advanced-stage ovarian cancer patients, with a higher value of this parameter being considered as a sign of the biological decline in ovarian cancer patients [17]. In the study conducted by Komura et al., the authors came to demonstrate the superiority of CRP/alb when compared to CRP alone in predicting the long-term outcomes of ovarian cancer patients [30]. Similarly to our study, Liu et al. demonstrated that a higher value of the CRP/alb ratio was associated with more advanced stages of the disease, with higher amounts of residual tumor, of ascites, with more elevated CA125 levels as well as with poorer GS and MGS [47].

When it comes to GS and MGS, we should underline the fact that both scores represent a combination of the preoperative values of CRP and albumin. Studies conducted on the issue of the prognostic role of GS and MGS in ovarian cancer patients so far have demonstrated that higher values of these parameters are associated with lower rates of complete debulking surgery and poorer rates of survival. Therefore, in the study conducted by Zhu et al. on 672 patients with advanced-stage ovarian cancer, the authors underlined the fact that higher preoperative GS and MGS are usually associated with incomplete debulking (*p* = 0.007), with undifferentiated tumors (*p* = 0.001), with poorer responses to neoadjuvant chemotherapy as well as with poorer disease-free and overall survival rates [48].

Therefore, the data presented in this paper come to underline the fact that nutritional inflammatory conditions represent significant prognostic factors for the postoperative outcomes of ovarian cancer patients. However, the main limitation of our study is related to the low number of cases considered to be eligible for being introduced in this analysis as well as by the retrospective character of this study. In order to better validate these results, larger, prospective studies are still needed.

Meanwhile, once these affirmations are widely accepted, they will create the premise of applying the concept of artificial intelligence in the field of advanced-stage ovarian cancer. Therefore, information obtained so far can be stored in electronic databases and can be used for creating prediction models in order to establish a profile of patients at risk of having a poorer outcome. Once this profile is created, it will be compared to data collected from the general population and will provide a safer and faster recognition of women at risk of developing ovarian cancer at a certain moment of their life [48,49,50]. Therefore, although the data published so far in the field of artificial intelligence and ovarian cancer were rather focused on high-grade serous ovarian cancer and were based on computed tomography images, which were stored and analyzed by artificial neural networks and specific algorithms, we are once more enabled to consider that the prognostic factors will be widely validated and that new prognostic models will be imagined [51,52,53].

## 5. Conclusions

A strong relationship between inflammation, nutritional status and the long-term outcomes of ovarian cancer patients seems to exist. In this respect, a preoperative determination of different scores, such as the CRP/albumin ratio, GS, MGS or PNI, represents a cornerstone in identifying the most suitable cases for per primam cytoreduction. Once cases have been identified as being at risk of having a poorer postoperative outcome, they should be excluded from per primam surgery and reorientated to neoadjuvant systemic therapy followed by imagistic restaging and surgical procedures. In our opinion, future research should target the validation of these prognostic scores through larger, prospective studies as well as the development of new models in order to improve the diagnostic and prognostic predictive abilities in advanced-stage ovarian cancer patients.

## Figures and Tables

**Table 1:** Glasgow score and modified Glasgow score were used in order to classify the inflammatory and nutritional status.

	CRP (mg/L)	Albumin (mg/L)	Value
Glasgow score	<10 mg/L	>35 g/L	0
	<10 mg/L	<35 g/L	1
	>10 mg/L	<35 g/L	2
Modified Glasgow score	<10 mg/L	>35 g/L	0
	<10 mg/L	<35 g/L	0
	>10 mg/L	<35 g/L	1

Abbreviations: CRP—C-reactive protein.

**Table 2:** The relation between CRP/alb ratio and demographic, intraoperative and postoperative parameters. The distribution of continuous variables was assessed by using the *t*-test.

Parameter/Number of Cases	CRP/Alb < 0.05 (n = 9 Cases)	CRP/Alb > 0.05 (n = 48 Cases)	*p*-Value
Age (years, mean)	48	53	0.091
Associated comorbidities			
- Yes	2 (22.2%)	14 (29.1%)	1
- No	7 (77.8%)	34 (70.9%)	
FIGO stage:			
- IIIC	7 (77.8%)	44 (91.6%)	0.232
- IV	2 (22.2%)	4 (8.4%)	
CA125 (U/dL)	1142	2825	0.003
Ascites (mL, mean value)	1292	3561	<0.001
PCI:			
<10	4 (44.4%)	7 (14.5%)	0.065
>10	5 (55.6%)	41 (85.4%)	
Histopathological subtype:			
- Seros	5 (55.6%)	4 (8.4%)	0.003
- Non-serous	4 (44.4%)	44 (91.6%)	
Differentiation degree:			
- G1, G2	5 (55.6%)	11 (23%)	0.098
- G3	4 (44.4%)	37 (77%)	
Maximum diameter of the tumor:			
- <5 cm	6 (66.6%)	16 (33.4%)	0.074
- >5 cm	3 (33.4%)	32 (66.6%)	
Lymphatic metastases:			
- Yes	2 (22.2%)	28 (58.3%)	0.071
- No	7 (77.8%)	20 (41.7%)	
NLR	1.75	4.71	<0.001
PLR	133	352	0.001
MLR	0.17	0.62	0.011
SII	519,920	1,989,540	0.004
Total serum protein (g/dL)	7.3	5.7	0.003
Hb (g/dL)	13.1	11.6	0.015
Extended upper abdominal resections			
- Yes	8 (88.9%)	32 (66.7%)	0.254
- No	1 (11.1%)	16 (33.3%)	
Postoperative complications:			
- Yes	0 (0%)	16 (33.3%)	0.049
- No	9 (100%)	32 (66.7%)	
Disease-free survival (mean, months)	9	6	0.019
Overall survival (mean, months)	20	9	0.01

Abbreviations: CRP—C-reactive protein; CRP/Alb—CRP/albumin; NLR—neutrophil to lymphocyte ratio; PLR—platelet to lymphocyte ratio; MLR—monocyte to lymphocyte ratio; SII—systemic inflammatory index; Hb—hemoglobin.

**Table 3:** The distribution of perioperative parameters according to the Glasgow scale.

Parameter No of Cases	GS0 (n = 19)	GS1 (n = 21)	GS2 (n = 17)	*p*-Value
Age (years, mean)	52.8	52.9	64.6	0.005
CA125 (U/dL)	547	2317	6223	<0.001
Ascites (mL, mean value)	1610	2009	3600	<0.001
NLR	2.1	3.6	7.3	<0.001
PLR	162	310	499	<0.001
MLR	0.24	0.45	1	<0.001
SII	634,924	1,383,884	3,532,475	<0.001
Total serum protein (g/dL)	7.1	6.3	4.3	<0.001
Hb (g/dL)	12.9	12.2	10.2	0.032
CRP/Albumin	0.07	0.3	0.93	<0.001
Disease-free survival (mean, months)	35	18	6	0.041
Overall survival (mean, months)	42	27	12	0.044

Abbreviations: GS—Glasgow score; CRP—C-reactive protein; NLR—neutrophil to lymphocyte ratio; PLR—platelet to lymphocyte ratio; MLR—monocyte to lymphocyte ratio; SII—systemic inflammatory index; Hb—hemoglobin.

**Table 4:** The relation between MGS and perioperative and postoperative outcomes in the studied group.

Parameter No of Cases	MGS			
	0 (n = 32)	1 (n = 8)	2 (n = 17)	*p*-Value
Age (years, mean)	51	58	64	0.023
CA125 (U/dL, mean)	1014	3324	6223	<0.001
Ascites (mL, mean)	1618	2625	3600	<0.001
NLR	2.6	4.13	7.36	<0.001
MLR	0.29	0.61	1	<0.001
SII	808,231	1,782,716	3,532,475	<0.001
CRP/albumin	0.14	0.41	0.94	0.023
Total serum protein (g/dL)	6.68	6.93	4.39	0.012
Hb (g/dL)	12.75	11.79	10.29	0.081
Disease-free survival (mean, months)	31	13	6	0.01
Overall survival (mean, months)	50	19	12	0.01

Abbreviations: MGS—modified Glasgow score; CRP—C-reactive protein; NLR—neutrophil to lymphocyte ratio; MLR—monocyte to lymphocyte ratio; SII—systemic inflammatory index; Hb—hemoglobin.

**Table 5:** The correlation between PNI and clinical and the biological parameters of the investigated group of patients.

Parameter/No of Cases	PNI = 0 (n = 18 Cases)	PNI = 1 (n = 39 Cases)	*p*-Value
Age (years, mean)	53	57	0.131
Associated comorbidities			
- Yes	1 (5.56%)	13 (33.3%)	0.045
- No	17 (94.4%)	26 (66.7%)	
FIGO stage:			
- IIIC	16 (88.9%)	35 (89.8%)	1
- IV	2 (11.1%)	4 (10.2%)	
CA125 (U/dL)	682	3912	0.003
Ascites (mL, mean)	1661	2669	0.295
PCI:			
<10	9 (50%)	2 (5.1%)	<0.001
>10	9 (50%)	37 (94.9%)	
Histopathological type:			
- Serous	3 (16.7%)	6 (15.4%)	1
- Non-serous	15 (83.3%)	33 (84.6%)	
Differentiation degree:			
- G1, G2	11 (61.1%)	6 (15.4%)	0.001
- G3	7 (38.9%)	33 (84.6%)	
Maximal diameter of the tumor:			
<5 cm	5 (27.8%)	17 (43.6%)	0.382
>5 cm	13 (72.2%)	22 (56.4%)	
Lymph node metastases:			
- Yes	3 (16.7%)	27 (69.2)	<0.001
- No	15 (83.3%)	12 (30.8%)	
NLR	1.99	5.28	0.001
PLR	128	401	0.001
MLR	0.2	0.7	0.008
SII	598,145	2,292,579	0.015
Total serum proteins (g/dL)	7.19	5.49	0.018
Hb (g/dL)	12.87	11.43	0.848
Upper abdominal resections:			
- Yes	10 (55.5%)	30 (76.9%)	0.126
- No	8 (44.5%)	9 (23.1%)	
Postoperative complications:			
- Yes	1 (5.5%)	15 (38.5%)	0.011
- No	17 (94.5%)	24 (61.5%)	
Disease-free survival (mean, months)	35	11	<0.001
Overall survival (mean, months)	54	21	<0.001

Abbreviations: PCI—peritoneal carcinomatosis index; NLR—neutrophil to lymphocyte ratio; MLR—monocyte to lymphocyte ratio; SII—systemic inflammatory index; Hb—hemoglobin.

## Data Availability

Data are available upon reasonable request.
